# Thymic Hyperplasia with Lymphoepithelial Sialadenitis (LESA)-Like Features: Strong Association with Lymphomas and Non-Myasthenic Autoimmune Diseases

**DOI:** 10.3390/cancers13020315

**Published:** 2021-01-16

**Authors:** Stefan Porubsky, Zoran V. Popovic, Sunil Badve, Yara Banz, Sabina Berezowska, Dietmar Borchert, Monika Brüggemann, Timo Gaiser, Thomas Graeter, Peter Hollaus, Katrin S. Huettl, Michaela Kotrova, Andreas Kreft, Christian Kugler, Fabian Lötscher, Burkhard Möller, German Ott, Gerhard Preissler, Eric Roessner, Andreas Rosenwald, Philipp Ströbel, Alexander Marx

**Affiliations:** 1 Institute of Pathology, University Medical Center of the Johannes Gutenberg University Mainz, Langenbeckstraße 1, 55101 Mainz, Germany; andreas.kreft@unimedizin-mainz.de; 2 Institute of Pathology, University Medical Centre Mannheim, University of Heidelberg, Theodor-Kutzer-Ufer 1-3, 68167 Mannheim, Germany; zoran.popovic@umm.de (Z.V.P.); Timo.Gaiser@umm.de (T.G.); alexander.marx@umm.de (A.M.); 3 Department of Pathology and Laboratory Medicine, Indiana University School of Medicine, Indianapolis, IN 46202, USA; sbadve@iupui.edu; 4 Institute of Pathology, University of Bern, Murtenstrasse 31, 3008 Bern, Switzerland; yara.banz@pathology.unibe.ch (Y.B.); sabina.berezowska@chuv.ch (S.B.); 5 Department of Surgery, Armed Forces Hospital, Scharnhorststr.13, 10115 Berlin, Germany; dietmar.borchert@doctors.org.uk; 6 Unit for Hematological Diagnostics, Medical Department II, University Medical Center Schleswig-Holstein, Langer Segen 8-10, 24105 Kiel, Germany; m.brueggemann@med2.uni-kiel.de (M.B.); m.kotrova@med2.uni-kiel.de (M.K.); 7 Department of Thoracic Surgery, Klinik Löwenstein, Geißhölzle 62, 74245 Löwenstein, Germany; thomas.graeter@klinik-loewenstein.de; 8 Department of Thoracic Surgery, Katholisches Klinikum Mainz, An der Goldgrube 11, 55131 Mainz, Germany; peter.hollaus@t-online.de; 9 Department of Clinical Pathology, Robert-Bosch-Krankenhaus, and Dr. Margarete Fischer-Bosch Institute for Clinical Pharmacology, Auerbachstraße 110-112, 70376 Stuttgart, Germany; Katrin.Huettl@rbk.de (K.S.H.); german.ott@rbk.de (G.O.); 10 Department of Thoracic Surgery, LungenClinic Großhansdorf, Wöhrendamm 80, 22927 Großhansdorf, Germany; c.kugler@lungenclinic.de; 11 Department of Rheumatology and Immunology, Inselspital, Universitätsspital Bern, Freiburgstrasse, 3010 Bern, Switzerland; fabian.loetscher@insel.ch (F.L.); burkhard.moeller@insel.ch (B.M.); 12 Department of Thoracic Surgery, Robert-Bosch-Krankenhaus, Klinik Schillerhöhe, Solitudestraße 18, 70839 Gerlingen, Germany; Gerhard.Preissler@klinik-schillerhoehe.de; 13 Academic Thoracic Center Mainz, Division of Thoracic Surgery, University Medical Center of the Johannes Gutenberg University Mainz, Langenbeckstraße 1, 55101 Mainz, Germany; Eric.Roessner@unimedizin-mainz.de; 14 Institute of Pathology, University of Würzburg, Josef-Schneider-Str. 2, 97080 Würzburg, Germany; rosenwald@uni-wuerzburg.de; 15 Institute of Pathology, University Medical Center Göttingen, University of Göttingen, Robert-Koch-Straße 40, 37075 Göttingen, Germany; philipp.stroebel@med.uni-goettingen.de

**Keywords:** autoimmune disease, imaging, LESA, lymphoma, myasthenia, pathology, surgery, thymus, thymic epithelial tumor, thymitis

## Abstract

**Simple Summary:**

Thymic epithelial tumors and lymphomas are the most frequent mediastinal mass lesions. Thymic hyperplasia with “lymphoepithelial sialadenitis (LESA)-like features” (LESA-like TH) was initially described as one form of thymic hyperplasia and was thought not to be associated with autoimmune and lymphoproliferative diseases. Our systematic analysis of patients with LESA-like TH shows that 14% have associated lymphomas and 33% partially overlapping autoimmune diseases. This implies a hematologic and rheumatologic workup in patients with LESA-like TH. In addition, LESA-like TH should enter the list of differential diagnoses of mediastinal mass lesions, in particular in patients with autoimmune diseases.

**Abstract:**

Thymic hyperplasia (TH) with lymphoepithelial sialadenitis (LESA)-like features (LESA-like TH) has been described as a tumor-like, benign proliferation of thymic epithelial cells and lymphoid follicles. We aimed to determine the frequency of lymphoma and autoimmunity in LESA-like TH and performed retrospective analysis of cases with LESA-like TH and/or thymic MALT-lymphoma. Among 36 patients (21 males) with LESA-like TH (age 52 years, 32–80; lesion diameter 7.0 cm, 1–14.5; median, range), five (14%) showed associated lymphomas, including four (11%) thymic MALT lymphomas and one (3%) diffuse large B-cell lymphoma. One additional case showed a clonal B-cell-receptor rearrangement without evidence of lymphoma. Twelve (33%) patients (7 women) suffered from partially overlapping autoimmune diseases: systemic lupus erythematosus (*n* = 4, 11%), rheumatoid arthritis (*n* = 3, 8%), myasthenia gravis (*n* = 2, 6%), asthma (*n* = 2, 6%), scleroderma, Sjögren syndrome, pure red cell aplasia, Grave’s disease and anti-IgLON5 syndrome (each *n* = 1, 3%). Among 11 primary thymic MALT lymphomas, remnants of LESA-like TH were found in two cases (18%). In summary, LESA-like TH shows a striking association with autoimmunity and predisposes to lymphomas. Thus, a hematologic and rheumatologic workup should become standard in patients diagnosed with LESA-like TH. Radiologists and clinicians should be aware of LESA-like TH as a differential diagnosis for mediastinal mass lesions in patients with autoimmune diseases.

## 1. Introduction

Thymic epithelial tumors (thymomas and thymic carcinomas) and lymphomas represent the two most frequent types of mediastinal neoplasms in adults [1]. Non-neoplastic mediastinal masses mainly consist of cysts and several types of thymic hyperplasia: (i) true thymic hyperplasia of unknown etiology that shows normal-looking histology and a thymus weight that mostly exceeds 100 g, (ii) rebound hyperplasia that follows the cessation of various “stressors” such as infection or chemotherapy and shows normal-for-age or juvenile histology and organ weights usually below 100 g, (iii) diseases with the unifying histological hallmark of lymphofollicular hyperplasia. In this group, the paradigmatic and most frequent disease is the “conventional thymitis” with lymphofollicular hyperplasia that typically occurs in early-onset myasthenia gravis (EOMG) and rarely other settings and is labelled here as “thymic follicular hyperplasia” (henceforth abbreviated as TFH). Recently, another condition characterized by lymphofollicular hyperplasia was described and named “thymic hyperplasia with lymphoepithelial sialadenitis (LESA)-like features” (henceforth called LESA-like TH) that forms mediastinal mass lesions with diameters between 4 and 22 cm in corticosteroid-naïve patients without MG [2,3]. Those masses were either detected fortuitously or due to complications following compression of the heart, great vessels and lung. The reported histological hallmarks included proliferation of epithelial cells and Hassall corpuscles, cystic changes, lymphoepithelial lesions and lymphofollicular hyperplasia leading to a replacement of the thymic parenchyma by numerous follicles and plasma cells with consecutive lymph node-like appearance. The five patients described so far did not show any autoimmune, immune mediated inflammatory or lymphoproliferative diseases, although such an association might have been anticipated in analogy to the “true” LESA of salivary glands [4]. Furthermore, the first descriptions of primary thymic mucosa-associated lymphoid tissue (MALT) lymphomas already highlighted the striking similarity with “myoepithelial sialadenitis” [5]. However, until now, systematic investigations into a relationship between LESA-like TH and lymphomas as well as autoimmune diseases have not been undertaken.

The aim of this study, therefore, was to analyze a large cohort of patients with LESA-like TH in terms of association with autoimmune disorders, lymphomas and histology. In addition, we screened cases primarily diagnosed as thymic MALT lymphomas for possible remnants of LESA-like lesions.

## 2. Materials and Methods

A retrospective analysis of reference pathology cases with LESA-like TH and thymic lymphomas was performed. The study was approved by the local ethics committee (Medical Ethics Committee II, Medical Faculty Mannheim of the Heidelberg University, approval no. 2017-806R-MA). For the sake of simplicity we counted asthma among the autoimmune diseases, although it is now considered an immune mediated inflammation. The tissue had been subjected to routine protocols of diagnostic pathology. Rearrangements of the B-cell receptor were tested by Biomed-2/EuroClonality multiplex PCR primers. Due to the limited sample quality, it was impossible to perform IGH-FR1 next generation sequencing [6] to characterize the lymphoma-associated rearrangements more closely. Immunohistochemistry was performed using diaminobenzidine (DAB) substrate chromogen detection system (Dako, Hamburg, Germany) and primary antibodies against IgA, IgD, IgG, IgM (polyclonal, Agilent, Frankfurt, Germany A0262, A0093, A0423, A0425), BCL2 (clone: 124), BCL6 (PGB6p), CD5 (4C7), CD10 (56C6), CD20 (L26), CD23 (SP23), CD30 (BerH2), MUM1/IRF4 (MUM1p), cytokeratin 19 (RCK108), desmin (polyclonal, Thermo Scientific, RB-9014P), kappa/lambda (polyclonal A0191, A0193 Agilent) and TdT (SEN28).

## 3. Results

### 3.1. Clinical Presentation

**Figure 1:**
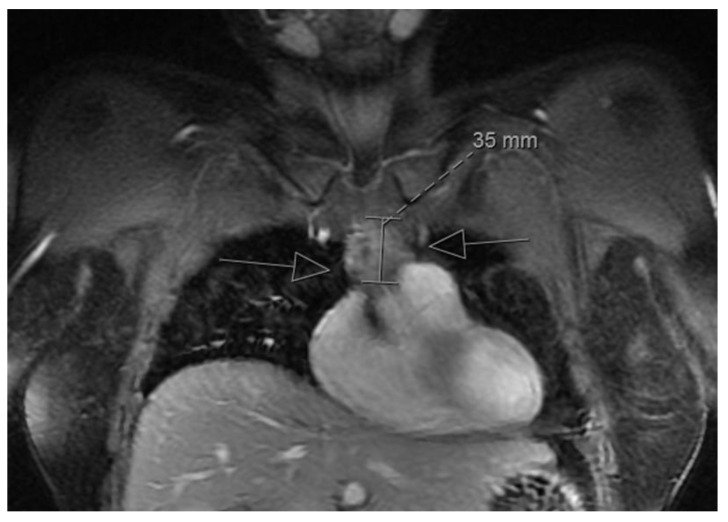
Magnetic resonance imaging in a LESA-like TH patient: Mediastinal mass-forming lesion with a diameter of 35 mm (arrows) in a patient with LESA-like TH (incidental finding).

The clinicopathological features of 36 cases of LESA-like TH submitted for reference pathology from 2011 to 2019 are given in Table 1. The male-to-female ratio was 1.4:1, and the mean age was 53 ± 12 (SD) years (range 32–80). In all but two cases (94%) the mass lesions were incidental findings in CT or MRI scans or during surgery for other reasons (e.g., bypass) (Figure 1). The two symptomatic patients were a 67-year-old man and a 32-year-old woman suffering from MG that was suspected to be thymoma-associated.

### 3.2. Histological Findings

**Figure 2:**
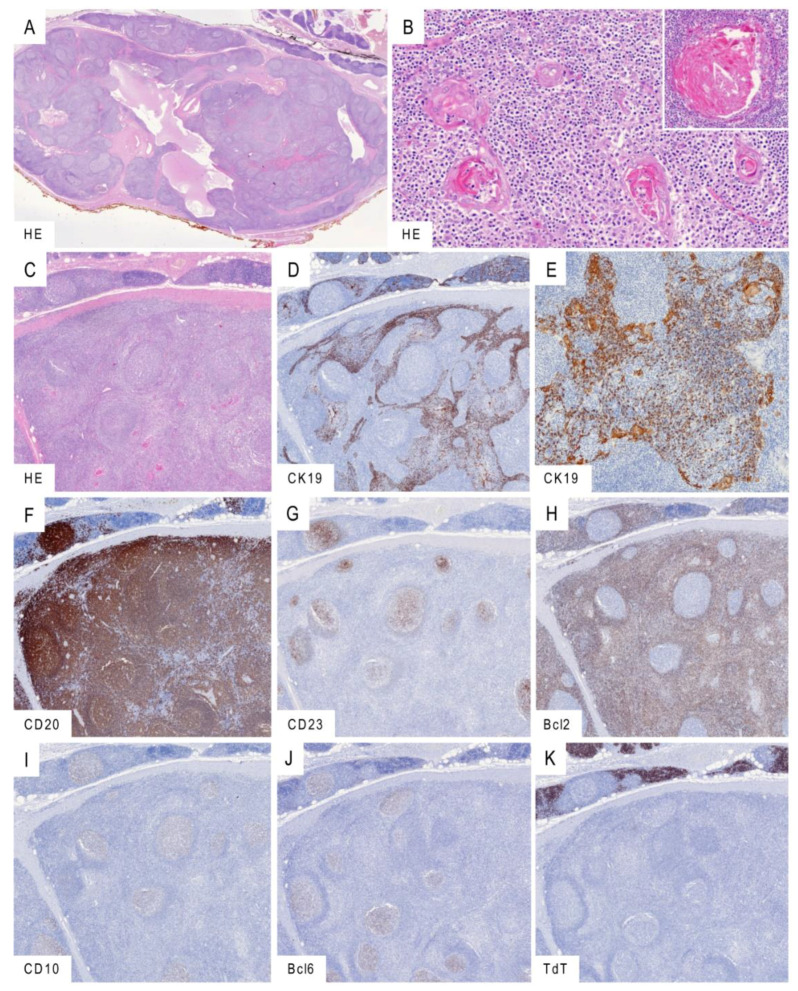
Histological findings in LESA-like TH. (**A**): Low-power magnification revealing lymph node-like change with typical cystic structures and remnants of thymus discernible in the upper right corner. (**B**): Epithelial hyperplasia with lymphocytic epitheliotropism and hyperplasia of Hassall corpuscles showing cystic dilatation (inlay). (**C**–**K**): Serial sections capturing the abrupt transition of moderate conventional thymitis (upper part) into the tumor-forming LESA-like TH (lower part) in conventional HE morphology (**C**) and immunohistochemistry for cytokeratin 19 visualizing the hyperplastic epithelium with intraepithelial lymphocytes (**D**,**E**). Dense B lymphocytic infiltration shown by CD20 (**F**) with prominent CD23-positive follicular dendritic cells (**G**) colonized by Bcl2-negative (**H**) and CD10/Bcl6-positive (**I**,**J**) lymphocytes. As contrasted with the moderate thymitis in the upper part, LESA-like TH is typically devoid of immature TdT-expressing T-lymphocytes (**K**). Micrographs taken from the LESA-like TH case no. 5. Original magnifications: (**A**): 1.5×; (B): 20×, inlay 30×; (**C**,**D**,**F**–**K**): 3×; (**E**): 15×.

Histology consistently revealed a tumor-forming process with a lymph node-like appearance (Figure 2A–C). Cysts lined by squamous epithelium with occasional cholesterol crystals were frequent. The lesions often did not involve the whole thymus but spared thymic lobes that showed mild to moderate “conventional” lymphofollicular hyperplasia. The medullary epithelium was hyperplastic and readily discernible as trabeculae and networks in immunostains for CK19, while in conventional HE stains its hyperplastic character was sometimes obscured by prominent lymphoid infiltrates that elicited lymphoepithelial lesions (Figure 2D,E). Hassall corpuscles were consistently maintained and often hyperplastic with a microcystic change. The lymphocytic infiltrate consisted of mature B-cells and T-cells with numerous germinal centers visualized by CD23(+) follicular dendritic cells (Figure 2F,G). In 31 cases (86%), hyperplastic germinal centers showed a typical population of CD20(+), Bcl2(−), Bcl6(+) and CD10(+) B-cells (Figure 2H–J). In contrast to unaffected lobes, those with full-blown LESA-like TH showed cortical atrophy with complete or subtotal absence of immature, TdT(+) T-cells (Figure 2K). The number of desmin(+) myoid cells was unremarkable.

Taking the number of mediastinal mass lesions referred for consultation to the Institute of Pathology, University Medical Centre Mannheim, Germany, into account, one might roughly estimate the frequency of LESA-like TH as approximately 2% (Table S1). However, due to the referral bias, the true incidence might rather be lower.

A concomitant thymoma was observed in only one patient. This micronodular thymoma with lymphoid stroma was widely separated from the LESA-like TH and showed features distinctly different from those of LESA-like TH: sharply delineated nodules of plump spindle epithelial cells, lack of Hassall corpuscles and a lymphoid stroma harboring immature, TdT(+) T-cells around the epithelial nodules in addition to mature B-cells and T-cells (Figure S1).

### 3.3. LESA-Like TH-Associated Lymphomas

**Figure 3:**
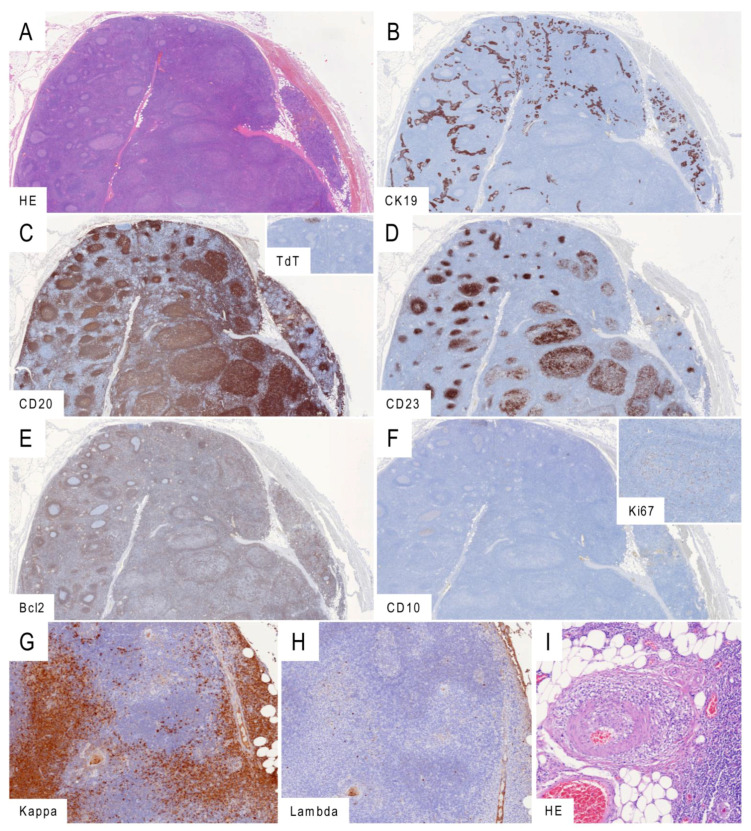
Marginal zone lymphomas associated with LESA-like TH. (**A**–**F**): Serial sections of a LESA-like TH (left) progressing into a marginal-zone lymphoma (right). The process is readily discernible as a rarefication of the cytokeratin 19-positive epithelium (**B**) and expansion of the CD20-positive lymphocytic component (**C**) with tiny marginal remnants of TdT-expressing cortical thymocytes (inlay). The transformation is characterized by atypical and hyperplastic meshworks of CD23-positive follicular dendritic cells (**D**) populated by Bcl2-positive (**E**) and CD10-negative lymphocytes (**F**) with an abnormally distributed and low Ki67-proliferation in the germinal centers (inlay). (**G**–**H**): Prominent secretory differentiation and light-chain restriction for kappa (**G**) in contrast to lambda (**H**) observed in one of the marginal zone lymphomas associated with LESA-like TH. (**I**): Vasculitis in the direct neighborhood of the thymus in the unique patient with simultaneous LESA-like TH, MALT lymphoma and autoimmunity (Sjögren syndrome, systemic lupus erythematosus, rheumatoid arthritis). Micrographs taken from the LESA-like TH cases no. 31 (**A**–**F**) and 29 (**G**–**I**). Original magnifications: (**A**–**F**): 1.5×, (**G**,**H**): 8×; (**I**): 10×.

In five of 36 patients primarily diagnosed with LESA-like TH (14%), an accompanying B-cell lymphoma was observed, including four MALT-lymphomas (11%) and one diffuse large cell B-cell lymphoma (DLBCL, 3%) (Table 1). An additional patient showed a monoclonal rearrangement of B-cell receptor genes but no morphological features of lymphoma. All five lymphomas developed inside or in continuity with the LESA-like TH (Figure 3 and Figure S2). Among the four MALT-lymphomas, three were restricted for IgG and one for IgM. The MALT-lymphomas showed enlarged meshworks of CD23(+) follicular dendritic cells colonized by CD20(+) Bcl2(+), Bcl6(−) and CD10(−) B lymphocytes (Figure 3A–H). In the case with simultaneous LESA-like TH, MALT lymphoma and autoimmunity (no. 29), a plasma cell-rich vasculitis was present (Figure 3I). The one LESA-like TH-associated DLBCL exhibited a germinal center phenotype with expression of CD20, BCL2, BCL6 and CD10, while typical markers of primary mediastinal large B-cell lymphoma (CD23, CD30, IRF4/MUM1) were missing.

Among 11 archival cases of primary thymic MALT lymphomas with a male-to-female ratio of 2:9 and a mean age of 61 ± 14 years (range 34–79), two cases (18%, one male and one female) showed remnants of LESA-like TH in a retrospective screen. Except for one female with Sjögren syndrome, these patients did not show autoimmune diseases. A heavy chain restriction could be documented by immunohistochemistry in five (two IgA, two IgG and one IgM) out of nine cases with material available for additional investigations (Table 2).

### 3.4. LESA-Like TH-Associated Autoimmune Disorders

Twelve patients (33%; five men and seven women) suffered from partially overlapping autoimmune diseases that were present already at the time of thymectomy: systemic lupus erythematosus (*n* = 4, 11%), rheumatoid arthritis (*n* = 3, 9%), myasthenia gravis (*n* = 2, 6%), asthma (*n* = 2, 6%), Sjögren syndrome, scleroderma, pure red cell aplasia, Grave’s disease and anti-IgLON5 syndrome (each *n* = 1, 3%) (Table 1). Of note, among the two patients with MG, one 68-year-old male patient who was suspected to have a thymoma prior to surgery showed high anti-titin and low anti-acetylcholine receptor (AChR) autoantibody titers, i.e., a spectrum of findings that is characteristic of “late-onset MG” (LOMG) in non-thymoma patients. The other MG patient, a 32-year-old female, showed anti-AChR antibodies. A third patient presented with non-myasthenic neurological deficits and showed anti-muscle-specific kinase (MuSK) antibodies.

**Figure 4:**
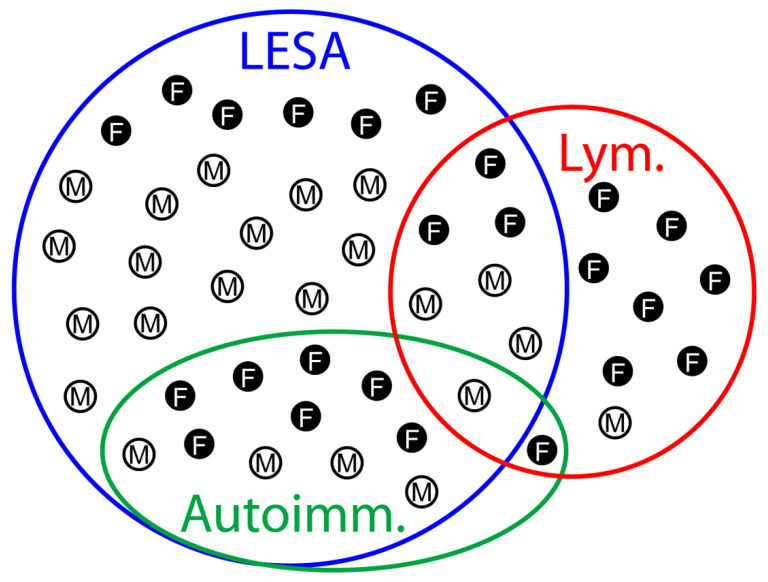
Venn diagram visualizing the overlaps between LESA-like TH, lymphomas and autoimmune diseases. A synopsis of both cohorts, i.e., LESA-like TH (Table 1) and lymphoma (Table 2), is shown with the overlaps between the diagnoses of LESA-like TH (LESA), lymphoma (Lym.) and autoimmune disorders (Autoimm.) together with the gender distribution (M, male; F, female). Six female and 14 male patients showed LESA-like TH only, while one male and seven female patients exclusively showed lymphoma. LESA-like TH overlapped with lymphomas and autoimmunity in three female and four male and seven female and five male patients, respectively, of whom one male suffered from all three diseases. One female showed autoimmunity and lymphoma without LESA-like TH.

Unexpectedly, the subgroups of patients with lymphomas and autoimmune diseases overlapped only in two cases: a 70-year-old male suffering from Sjögren syndrome, systemic lupus erythematosus, rheumatoid arthritis and thymic MALT lymphoma in the setting of a LESA-like TH and a 63-year-old female suffering from Sjögren syndrome and thymic MALT lymphoma without LESA-like TH (Table 1 and Table 2 and Figure 4).

## 4. Discussion

Based on the largest cohort of patients to date, we report on two clinically and diagnostically important new features observed in LESA-like TH that distinguish it from ”conventional” TFH: the association with lymphoma and mainly non-myasthenic autoimmune diseases, and the near total absence of thymic cortical structures even in corticosteroid-naïve patients.

These current new findings have the following implications for patient care: First, the classification of LESA-like TH as benign should be complemented by the fact that more than 10% of patients have a concomitant lymphoma at the time of surgery. Therefore, the pathological workup of thymectomy specimens must not only exclude thymoma but also lymphoma. To pursue this workflow, it is important not to misconceive LESA-like TH as unusually large but “innocent” TFH; recognition of a missing thymic cortex as a hallmark of LESA-like TH aids in this differential. Second, postoperative staging procedures in case of LESA-like TH-associated lymphomas appear mandatory like in other lymphoma settings, although dissemination requiring chemotherapy was restricted to the one DLBCL in our cohort. Third, since the course of LESA-like TH-associated lymphomas after surgery and the impact of radical surgery on the risk of lymphoma recurrence are unknown, long-term clinical follow-up is important, in order to learn whether adjuvant therapy may be beneficial after surgery. Fourth, radiologists and clinicians should be aware of LESA-like TH as a potential—albeit rare—differential diagnosis for mediastinal mass lesions in patients with autoimmune diseases. Fifth, clinical follow-up is also required to determine whether LESA-like TH resection might ameliorate the associated autoimmune disorders as thymectomy ameliorates EOMG [7]. In our cohort, this was not the case, but drawing conclusions may not yet be justified due to low case numbers. Nevertheless, we anticipate that the many contrasting features of LESA-like TH and TFH will make their further clinical, immunological and molecular comparison a promising scientific endeavor with the perspective to better understand the thymic function, the pathogenesis of MG and non-myasthenic autoimmune diseases and the impact of peripheral tolerance failure on the thymus.

Since the age of the lymphoma-free patients with LESA-like TH in the current series averaged 52 years, while the average age of patients with thymic MALT lymphoma was slightly higher (58 and 56 years, in our and other studies, respectively [8]), we hypothesize that LESA-like TH could be a precursor lesion in at least a subset of thymic MALT lymphomas. This idea is supported by the close spatial relationship between LESA-like TH and the lymphomas and the detection of remnants of LESA-like TH in two of 11 thymic MALT lymphomas in an independent cohort (Figure 3 and Figure S2). LESA-like TH-associated MALT lymphomas were peculiar in several respects: First, they did not show the preferred IgA-restriction that was observed in 13 out of 15 thymic MALT lymphomas reported from Japan and China [9]. Second, 11 of those 15 MALT lymphomas were associated with autoimmune diseases, while in our cohorts, only one of four patients with LESA-like TH-associated MALT lymphoma and one of 11 MALT-lymphomas showed autoimmune phenomena (Figure 4). Third, Sjögren syndrome, which occurred in eight of the 15 patients with thymic MALT lymphomas and is the leading disease underlying myoepithelial sialadenitis-associated lymphomas [10], was encountered in only one patient with LESA-like TH and in only one patient suffering from a MALT lymphoma without LESA-like TH. Fourth, 12 of the 15 MALT lymphoma patients in Inagaki et al.’s cohort [9] (including 10 of 11 MALT lymphoma patients with associated autoimmunity) were females, while the bias in our series was less pronounced (Figure 4). Whether these discrepancies reflect ethnic differences or the absence of underlying LESA-like TH in the cases from Japan and China cannot be decided. Similarly, it is unclear whether the association between the one DLBCL with LESA-like TH is fortuitous. Although no residual MALT lymphoma component was present in this case, the possibility of a high-grade transformation of thymic MALT lymphoma has been reported previously [8]. Primary salivary gland high-grade B-cell lymphomas occurring with or without a preceding MALT lymphoma have also been observed in Sjögren syndrome [11,12].

It is unclear why LESA-like TH (similarly to micronodular thymoma with lymphoid stroma) apparently predisposes to MALT lymphoma development while TFH in MG patients does not [13]. In micronodular thymoma with lymphoid stroma, the micromilieu differs substantially from the one in TFH and is therefore suspected to interfere with B-cell homoeostasis [13]. A similar scenario could be operative in LESA-like TH, as suggested by a key histological difference between TFH and LESA-like TH: while TFH is compatible with thymopoiesis as indicated by well-developed, TdT(+) cortical areas, LESA-like TH lacks cortical structures even in corticosteroid-naïve patients (Figure 2). This feature of LESA-like TH mimics the cytokine-induced abrogation of the thymic cortex and thymopoiesis in mouse models [14,15] and in humans during severe infections [16,17].

Another unresolved puzzle is the high prevalence of non-myasthenic autoimmune diseases and the paucity of MG in patients with LESA-like TH, while the opposite is typical of patients with either EOMG or thymoma. Whereas the frequencies of pure red cell aplasia, scleroderma and Graves’ disease in LESA-like TH (present in one patient each, approx. 3%) were comparable with previous reports on thymoma and EOMG patients [18], the prevalence of systemic lupus erythematosus (SLE) (*n*=4, 11%) and rheumatoid arthritis (RA) (*n* = 3, 8%) was remarkably high in the LESA-like TH cohort as contrasted with levels of 1–3% in thymomas [18,19] and EOMG [20]. The true incidence of autoimmune diseases might be even higher in our cohort because in older cases (listed at the top of the chronologically arranged Table 1), the clinical data were partially incomplete with regard to autoimmunity. Furthermore, the clinical symptoms of Sjögren syndrome, which is characterized by LESA of the lacrimal and salivary glands and frequently associated with RA and SLE, are often less apparent and may with only one case be underdiagnosed in this cohort. The pathogenesis of non-myasthenic autoimmune diseases associated with LESA-like TH is unknown, echoing the poor understanding of their pathogenesis in patients with EOMG and thymomas [21,22,23]. Likewise, it is unclear why MG is so rare in LESA-like TH. A different genetic background is a possible explanation, since only one patient with anti-AChR positive MG was encountered, and the other two LESA-like TH patients with MG-associated autoantibodies did not suffer from EOMG: One patient at age 67 had non-thymomatous, anti-titin/anti-AChR autoantibody-positive late-onset MG (LOMG), while the other patient (without overt MG symptoms) had anti-MuSK antibodies. Apart from these two patients, LOMG and anti-MuSK-MG typically do not emerge in the context of thymic pathology but have been thought to reflect a loss of peripheral tolerance [24,25]. Unfortunately, lack of blood or fresh material in all of these consultation cases precluded HLA-typing. Another mechanism that could underlie the rarity of MG in LESA-like TH is the consistent, near complete absence of thymopoiesis in LESA-like TH; this property is held responsible for the low incidence of MG in type A and metaplastic thymomas and thymic carcinomas [26], while thymopoiesis in thymomas and rare non-thymic tumors is strongly linked to MG development [23,27].

The main limitation of this study is the relatively small number of cases. Although the number of analyzed LESA-TH cases is unprecedented, it is too low to deduce reliable frequencies of lymphoproliferative and autoimmune diseases in these patients. The retrospective character of the analysis that is almost only based on consultation cases hampered the retrieval of clinical information and follow-up data.

In view of these facts, prospective studies are needed to (1) assess the incidence and full spectrum of autoimmune diseases and lymphomas in LESA-like TH patients; (2) to learn whether thymectomy has an impact on the autoimmune symptoms of patients with LESA-like TH and (3) to elucidate the clinical behavior of LESA-like TH-associated lymphomas following thymectomy. Moreover, a thorough comparison of molecular and genetic features (including genetic backgrounds) of LESA-like TH and ”conventional” TH appears highly promising to decipher the aberrant immunoregulatory mechanisms that underlie the high risk to develop mainly non-myasthenic autoimmunity and lymphomas in the former as compared to mainly MG development and protection from lymphomas in the latter.

## 5. Conclusions

In summary, the above findings show that LESA-like TH is not just an “exaggerated”, tumor-forming variant of TFH but, rather, a disease of its own standing with a remarkably high incidence of associated autoimmune diseases and lymphomatous transformation. These new findings argue for an in-depth workup of LESA-like TH resection specimens and a meticulous clinical management of LESA-like TH patients. Moreover, radiologists and clinicians should be aware of LESA-like TH as a differential diagnosis for mediastinal mass lesions in patients with autoimmune diseases.

## Figures and Tables

**Table 1:** Clinical data of cases primarily diagnosed with LESA-like TH.

Case	Age (y)	Sex	Size (cm)	Autoimmune Disorder	Lymphoma
1	57	f	7.0		
2	70	m	7.0		clonal JH rearrangement but no lymphoma
3	47	m	5.0		
4	62	f	n.r.		thymic MALT lymphoma (IgG)
5	50	m	3.5		
6	45	m	6.5		
7	65	f	8.5		
8	38	m	n.r.	Grave’s disease	
9	57	m	n.r.		
10	55	m	n.r.		
11	51	m	n.r.		
12	47	m	8.5		
13	54	f	1.0	pure red cell aplasia	
14	44	f	n.r.		
15	45	m	n.r.		
16	80	m	3.5		
17	51	f	9.0	rheumatoid arthritis, asthma	
18	52	m	n.r.		
19	43	f	8.0		
20	54	f	n.r.		
21	62	m	4.0	rheumatoid arthritis	
22	37	f	7	anti-phospholipid syndrome, suspicious for SLE, neurologic deficits not typical for MG but MuSK-positive	
23	42	m	5.4		
24	60	f	4.5	asthma	
25	39	f	5.6		thymic MALT lymphoma (IgG)
26	35	f	9.0		
27	67	f	n.r.	SLE	
28	61	m	14.5		
29	70	m	10.0	Sjögren syndrome, SLE, rheumatoid arthritis	thymic MALT lymphoma (IgM)
30	32	f	7.0	MG (AChR-positive; MuSK, titin-negative)	
31	35	m	n.r.		thymic MALT lymphoma (IgG)
32	67	m	7.0	MG (titin-positive, AChR low)	
33	47	f	12.0	SLE, scleroderma	
34	65	m	6.0		diffuse large B-cell lymphoma (n.hcr.)
35	66	m	n.r.	anti-IgLON5-Syndrom	
36	39	m	17		

AChR, acetylcholine receptor; MALT, mucosa-associated lymphatic tissue; MG, myasthenia gravis; n.hcr., no heavy chain restriction detectable in immunohistochemistry; n.r., not reported; SLE, systemic lupus erythematosus.

**Table 2:** Clinical data of cases primarily diagnosed with thymic lymphoma.

Case	Age (y)	Sex	Size (cm)	Autoimmune Disorders	LESA-like TH	Lymphoma
1	34	f	n.r.			MALT lymphoma (IgG)
2	62	m	>10			MALT lymphoma (n.m.)
3	44	f	4.0		present	MALT lymphoma (IgA)
4	72	f	n.r.			Composite MALT and mantle-cell lymphoma (IgM)
5	61	f	n.r.			MALT lymphoma (n.m.)
6	49	f	n.r.			MALT lymphoma (n.m.)
7	75	m	n.r.		present	MALT lymphoma (n.hcr.)
8	52	f	4.5			MALT lymphoma (IgG)
9	79	f	biopsy			MALT lymphoma (n.hcr.)
10	78	f	1.2			MALT lymphoma (n.hcr.)
11	63	f	biopsy	Sjögren syndrome		MALT lymphoma (IgA)

n.m., no material for additional testing; n.hcr., no heavy chain restriction detectable in immunohistochemistry; n.r., not reported.

## Data Availability

The data presented in this study are available in this article (and supplementary material).

## Supplementary Materials

The following are available online at https://www.mdpi.com/2072-6694/13/2/315/s1, Figure S1: Micronodular thymoma with lymphoid hyperplasia; Figure S2: Diffuse large B-cell lymphoma associated with LESA-like TH; Table S1: Frequency of reference cases of selected mediastinal lesions reviewed per year at the Institute of Pathology, University Medical Centre Mannheim, Germany.
