# Supplementary material for: Thymic Hyperplasia with Lymphoepithelial Sialadenitis (LESA)-Like Features: Strong Association with Lymphomas and Non-Myasthenic Autoimmune Diseases
Source: Cancers (Basel). 2021 Jan 16;13(2):315. doi: 10.3390/cancers13020315 (PMC7830871; doi:10.3390/cancers13020315)
Supplement: Supplementary file 1 [file cancers-13-00315-s001.pdf]

cancers

MDPI

Supplementary Materials: Thymic Hyperplasia with Lymphoepithelial Sialadenitis (Lesa)-Like Features: Strong Association with Lymphomas and Non-Myasthenic Autoimmune Diseases

Stefan Porubsky, Zoran V. Popovic, Sunil Badve, Yara Banz, Sabina Berezowska, Dietmar Borchert, Monika Brüggemann, Timo Gaiser, Thomas Graeter, Peter Hollaus, Katrin S. Huettl, Michaela Kotrova, Andreas Kreft, Christian Kugler, Fabian Lötscher, Burkhard Möller, German Ott, Gerhard Preissler, Eric Roessner, Andreas Rosenwald, Philipp Ströbel, Alexander Marx

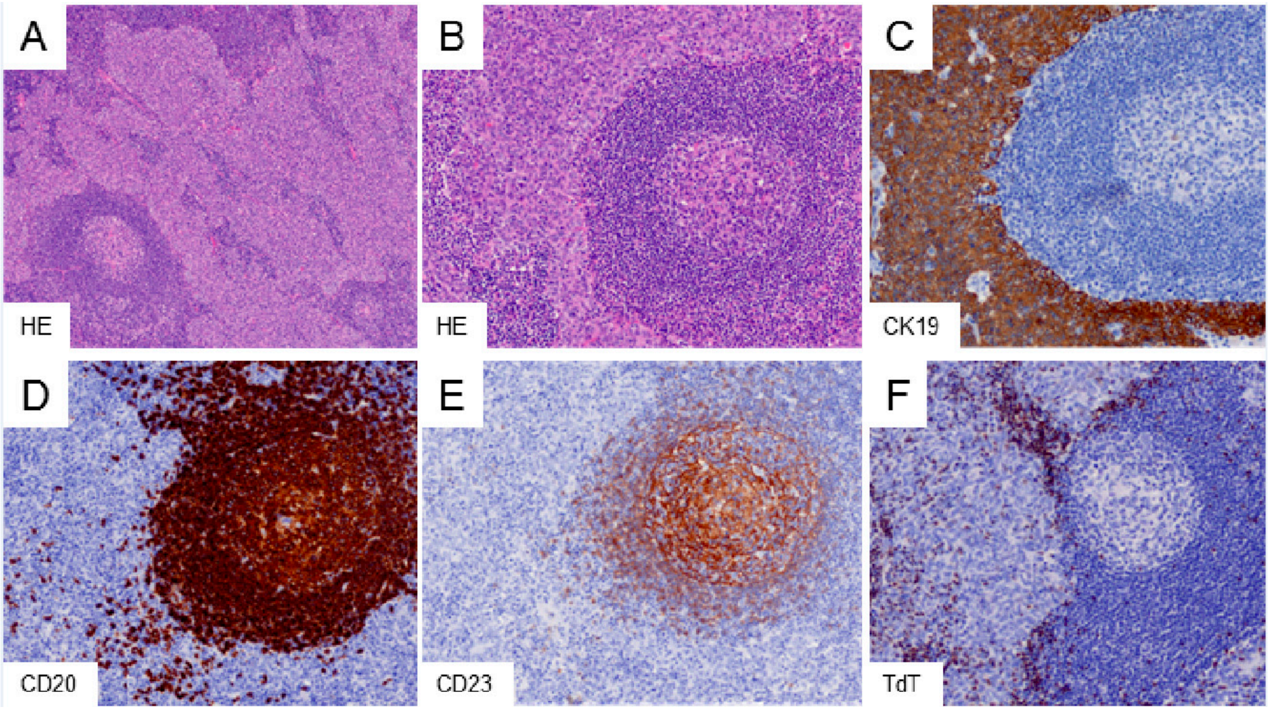

Figure S1. Micronodular thymoma with lymphoid hyperplasia. (**A**,**B**): Epithelial sheets separated by lymphoid stroma with follicles. In contrast to LESA-like TH, the epithelial cells are oval, densely packed and without overt squamous differentiation or Hassall corpuscles. (**C**,**D**) The cytokeratin 19-positive epithelial component (**C**) is sharply separated from the CD20-positive lymphoid stroma (**D**) with only few intraepithelial B-cells. (**E**) CD23-positive follicular dendritic cells may be numerous. (**F**) In contrast to LESA-like TH, immature TdT-expressing T-lymphocytes are typically present in low numbers in the epithelium and along its margins. Micrographs taken from the LESA-like TH case no.12. Original magnification: (**A**): 10×, (**B**–**F**): 20×.

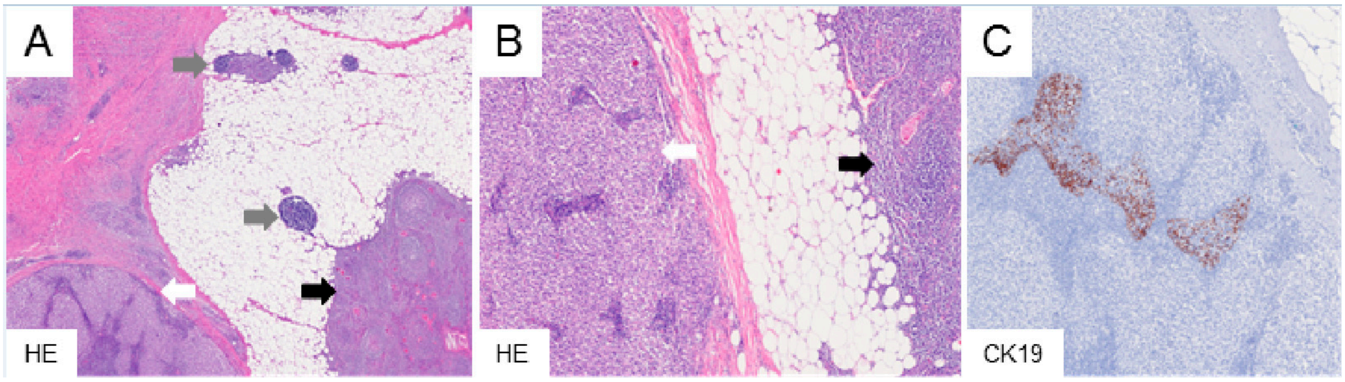

Figure S2. Diffuse large B-cell lymphoma associated with LESA-like TH. (**A**) and (**B**): Thymic remnants (gray arrows) and LESA-like TH (black arrow) in association with a sclerosing high-grade lymphoma (white arrow). (**C**) CK19 positive thymic epithelium enclosed in the high-grade lymphoma. Micrograhs taken form LESA-like TH case no. 34. Original magnification: (**A**): 2×, (**B**,**C**): 5×.

Table S1. Frequency of reference cases of selected mediastinal lesions reviewed per year at the Institute of Pathology, University Medical Centre Mannheim, Germany.

Diagnosis	Cases Per Year	Relative Frequency in %
Thymoma	150	79.79
Thymic carcinoma	20	10.64
TFH	6	3.19
Non-Hodgkin B-cell lymphoma	5	2.66
**LESA-like TH**	**4**	**2.13**
Thymolipoma	2	1.06
True thymic hyperplasia	1	0.53

As the low case numbers of thymic follicular hyperplasia (TFH) and lymphomas suggest, the frequencies are not representative of incidence values but reflect a strong referral bias.
